# Changes in prevalence and perinatal outcomes of congenital hydrocephalus among Chinese newborns: a retrospective analysis based on the hospital-based birth defects surveillance system

**DOI:** 10.1186/s12884-017-1603-2

**Published:** 2017-12-04

**Authors:** Ling Yi, Chaomin Wan, Changfei Deng, Xiaohong Li, Kui Deng, Yi Mu, Jun Zhu, Qi Li, Yanping Wang, Li Dai

**Affiliations:** 1 National Center for Birth Defects Monitoring, West China Second University Hospital, Sichuan University, Sec.3 No.17, South RenMin Road, Chengdu, Sichuan 610041 China; 2 Pediatric Department, West China Second University Hospital, Sichuan University, Chengdu, Sichuan China

**Keywords:** Congenital hydrocephalus, Birth defects, Prevalence, Perinatal outcome, Newborns

## Abstract

**Background:**

Little is known about the epidemiology of congenital hydrocephalus (CH) in China. This study aimed to depict recent changes in CH prevalence and perinatal outcomes of the affected newborns.

**Methods:**

Data were obtained from the Chinese Birth Defects Monitoring Network (CBDMN), which collects demographic information on all newborns above 28 weeks of gestation, and clinical information on neonates with congenital anomalies. CH cases delivered during 2005**–**2012 were analyzed. Poisson regression was used to calculate prevalence ratios (PR) and 95% confidence intervals, and linear chi-square test was used to examine time trend of CH prevalence.

**Results:**

Five thousand two hundred forty-five isolated and 1245 associated CH cases were identified among 10,574,061 newborns, yielding the prevalence of 4.96, 1.18 and 6.14 per 10,000 births for the isolated, associated and overall hydrocephalus, respectively. The annual prevalence of CH presented a decreasing trend (from 7.52 to 5.98 per 10,000 births, *P* < 0.001). Higher prevalence was found in both younger (<20 years, PR: 1.81, 95% CI: 1.56–2.10) and older (≥35 years, PR: 1.48, 95% CI: 1.36–1.61) maternal-age groups in comparison with the maternal-age group of 20 to 24 years. Higher prevalence was also found in infants born to mothers resided in rural areas, male infants, and multiple births. Of non-aborted infants with congenital hydrocephalus, 38.11% were born with low birth weight, 37.53% were preterm birth, and 20.69% died within 7 days after birth.

**Conclusions:**

Our findings present a relatively high prevalence and poor perinatal outcomes of CH in China, which can serve as a baseline for future study.

## Background

Congenital hydrocephalus (CH) is a group of neurological disorders caused by various reasons that all result in imbalance between the production and absorption of cerebrospinal fluid (CSF) [1]. The main characteristics are the accumulation of CSF in the cranial cavity and ventricular dilatation [2]. Besides a strong genetic background, prematurity, infection, and intracranial structural malformations such as neural tube defects (NTDs) and aqueduct stenosis can give rise to CH [3–6]. CH resulting from brain anomalies always have poor prognosis, particularly high neonatal fatality rate and impaired mental development. Although surgical interventions are widely used to improve the health of the affected children, life-long treatment or multidisciplinary care are needed. The prevalence of CH varied between 4 and 12 per 10,000 births [7–13]. The reported prevalence of CH in provinces of China ranged from 6.9 to 9.2 per 10,000 births [11–13]. Based on data from the Chinese Birth Defects Monitoring Network (CBDMN), an overall prevalence rate of 7.03/10,000 and an upward trend were observed during 1996–2004 [14]. Health care services for women and children have been improved considerably in China over the recent decade [15]. In particular, lots of medical institutions have been established to provide prenatal screening and diagnosis services for pregnant women since the *National Regulation on the Administration of Prenatal Diagnosis Techniques* took effect in 2003 [16], which led to the improvement of prenatal diagnosis and an increase in termination of pregnancies with severe birth defects. These changes had a great impact on the birth prevalence of certain birth defects in China, such as NTDs and Down syndrome [17, 18]. However, whether CH prevalence was affected by changes of the policy remains unknown. This study aimed to investigate recent changes in the prevalence of CH and perinatal outcomes of the affected neonates based on newly updated CBDMN database.

## Methods

### Study subjects

Data on CH cases and newborns in the study were abstracted from CBDMN, a nationwide hospital-based birth defects surveillance program in China, with monitoring period from 28 weeks of gestation to 7 days after birth. The procedure of data collection, case ascertainment and data quality management has been described in detail elsewhere [19, 20]. In brief, a three-level (county, province, and central) surveillance network and corresponding expert groups were established to perform routine data collection. Summary data of all births (live or stillbirths ≥ 28 weeks of gestation) and information of individual birth defect cases were collected with standardized forms by hospital staff and then checked by expert groups at each level. In addition, an independent retrospective survey was organized to identify underreporting of birth defects and inaccuracies in data, and database was subsequently updated prior to annual reporting. CBDMN adopts the same criteria of CH cases as described by the International Clearinghouse for Birth Defects Surveillance and Research (ICBDSR) [21]. CH cases characterized by dilatation of the cerebral ventricles and diagnosed prenatally or within the first week of life were included, and cases caused by premature birth, intraventricular haemorrhage or secondary to NTDs were excluded. We searched the CBDMN database from January 2005 to December 2012 for all births with a diagnosis of hydrocephalus, with any of the following ICD-10 codes were included in the study: Malformations of the aqueduct of Sylvius (Q03.0); Atresia of foramina of Magendie and Luschka, also called Dandy-Walker syndrome (Q03.1); other specified types of congenital hydrocephalus (Q03.8); and unspecified congenital hydrocephalus (Q03.9). Based on whether accompanied by additional malformations in other systems or organs, CH cases were divided into two subgroups: isolated CH and associated CH [7, 9].

Maternal age (<20, 20–24, 25–29, 30–34, ≥35 years), residential area (urban, rural), gender, and plurality of pregnancy were obtained for all births and CH cases. Residential areas were classified into urban (cities and towns) and rural (villages and countryside) areas based on mother’s last residence address where she lived for at least 1 year [20]. Birth weight (<2500, 2500–3999, ≥4000 g), gestational age (28–36, 37–41, ≥42 weeks), perinatal outcomes (stillbirth, early neonate death and alive within 7 days), and time of diagnosis were acquired for CH cases.

### Statistical analysis

The prevalence of CH was calculated as the number of CH cases, divided by the total number of live birth and stillbirth in CBDMN database during the 8-year period of the study. The changes in prevalence over the study period were analyzed by linear chi-square test [22]. CH prevalence stratified by maternal age, residential area, gender and plurality of pregnancy were compared by calculating prevalence ratios (PR) and 95% confidence intervals (95% CI) with Poisson regression model. All statistical analyses were performed with SPSS 21. The statistical significance level for α was set at 0.05.

## Results

**Fig. 1:**
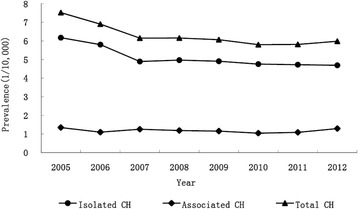
Trends in Prevalence of Congenital Hydrocephalus in China, 2005–2012

During the study period, a total of 6490 CH cases (5245 isolated and 1245 associated cases) were identified among 10,574,061 births, yielding a prevalence of 6.14, 4.96, and 1.18 per 10,000 births for the overall, isolated, and associated CH, respectively. There was a downward trend in the annual prevalence of overall CH (from 7.52 to 5.98 per 10,000 births, *P* < 0.001) and isolated CH (from 6.17 to 4.19 per 10,000 births, *P* < 0.001), but not in the prevalence of associated CH (Fig. 1).

The prevalence of isolated and associated CH varied significantly between groups stratified by maternal age, residential area, gender and plurality of pregnancy (Table 1). Both younger and older maternal age were associated with higher CH prevalence as compared to the maternal age group of 20–24 years. The highest prevalence of isolated CH was observed in maternal age < 20 years group (PR: 1.88, 95% CI: 1.60–2.22), whereas the highest prevalence of associated CH was in ≥35 years maternal age group (PR: 1.98, 95% CI: 1.67–2.36). Higher prevalence was also found in infants born to mothers who resided in rural areas (PR: 1.40, 95% CI: 1.34–1.48), infants with male sex (PR: 1.12, 95% CI: 1.06–1.17), and multiple births (PR: 2.55, 95% CI: 2.25–2.90).

**Table 1:** Prevalence of congenital hydrocephalus (1/10,000) in China during 2005–2012, stratified by maternal age, residential area, gender, and plurality of pregnancy

	Number of Births	Isolated CH (*N* = 5245)			Associated CH (*N* = 1245)			Total CH (*N* = 6490)		
		Number	Prevalence (95%CI)	PR (95%CI)	Number	Prevalence (95%CI)	PR (95%CI)	Number	Prevalence (95%CI)	PR (95%CI)
Maternal age (years)^a^										
< 20	196,160	155	7.90 (6.66–9.15)	**1.88 (1.60–2.22)**	29	1.48 (0.94–2.02)	**1.50 (1.03–2.18)**	184	9.38 (8.02–10.74)	**1.81 (1.56–2.10)**
20–24	2,764,522	1677	6.07 (5.78–6.36)	**1.45 (1.35–1.54)**	314	1.14 (1.01–1.26)	**1.15 (1.00–1.33)**	1991	7.20 (6.89–7.52)	**1.39 (1.31–1.48)**
25–29	4,435,764	1863	4.20 (4.01–4.39)	1.00 (ref)	438	0.99 (0.89–1.08)	1.00 (ref)	2301	5.19 (4.98–5.40)	1.00 (ref)
30–34	2,233,043	1008	4.51 (4.24–4.79)	**1.08 (1.00–1.16)**	279	1.25 (1.10–1.40)	**1.27 (1.09–1.47)**	1287	5.76 (5.45–6.08)	**1.11 (1.04–1.19)**
≥35	944,572	540	5.72 (5.23–6.20)	**1.36 (1.24–1.50)**	185	1.96 (1.68–2.24)	**1.98 (1.67–2.36)**	725	7.68 (7.12–8.23)	**1.48 (1.36–1.61)**
Residential area^b^										
Urban	5,630,037	2323	4.13 (3.96–4.29)	1.00 (ref)	582	1.03 (0.95–1.12)	1.00 (ref)	2905	5.16 (4.97–5.35)	1.00 (ref)
Rural	4,944,024	2920	5.91 (5.69–6.12)	**1.43 (1.36–1.51)**	662	1.34 (1.24–1.44)	**1.30 (1.16–1.45)**	3582	7.25 (7.01–7.48)	**1.40 (1.34–1.48)**
Gender^c^										
Female	4,940,848	2323	4.70 (4.51–4.89)	1.00 (ref)	513	1.04 (0.95–1.13)	1.00 (ref)	2836	5.74 (5.53–5.95)	1.00 (ref)
Male	5,630,497	2906	5.16 (4.97–5.35)	**1.10 (1.04–1.16)**	703	1.25 (1.16–1.34)	**1.20 (1.07–1.35)**	3609	6.41 (6.20–6.62)	**1.12 (1.06–1.17)**
Plurality of pregnancy^d^										
Singleton	10,412,509	5051	4.85 (4.72–4.98)	1.00 (ref)	1190	1.14 (1.08–1.21)	1.00 (ref)	6241	5.99 (5.85–6.14)	1.00 (ref)
Multiple	161,552	192	11.88 (10.20–13.57)	**2.45 (2.12–2.83)**	55	3.40 (2.50–4.30)	**2.98 (2.27–3.91)**	247	15.29 (13.38–17.20)	**2.55 (2.25–2.90)**

Abbreviations: *CI* Confidence interval, *PR* Prevalence ratio, *Ref* Reference

^a^2 cases with unspecified maternal age were excluded; ^b^3 cases with unspecified residential area were excluded; ^c^40 cases with unknown gender and 5 cases with unspecified gender were excluded; ^d^2 cases with unspecified plurality of pregnancy were excluded

Values in bold represent statistical significance at a 95% confidence level

Among 6490 CH cases, 5737 (88.47%) cases were diagnosed prenatally, 4942 (76.15%) cases were terminations of pregnancy (TOP). Characteristics of 1548 non-aborted CH cases were shown in Table 2, the low birth weight rate of associated CH was higher than that of isolated CH (45.98% vs 35.82%). Of infants affected by CH, 37.53% were born prematurely. The total stillbirth rate of CH was 34.35%. The early neonatal mortality rates (ENMR) were 17.93% (135/753), 28.63% (75/262) and 20.69% (210/1015) for the isolated, associated and overall hydrocephalus, respectively. ENMR of CH in rural area (141/578, 24.39%) was significantly higher than that in urban area (69/437, 15.79%).

**Table 2:** Characteristics of 1548 non-aborted cases with congenital hydrocephalus

	Isolated CH (*N* = 1201)		Associated CH (*N* = 347)		Total CH (*N* = 1548)	
	Number	Percent	Number	Percent	Number	Percent
Birth weight (g)^a^						
< 2500	428	35.82	160	45.98	588	38.11
2500–3999	705	59.00	173	49.71	878	56.90
≥4000	62	5.19	15	4.31	77	4.99
Gestational age (weeks)						
28–36	447	37.22	134	38.62	581	37.53
37–41	724	60.28	204	58.79	928	59.95
≥42	30	2.50	9	2.59	39	2.52
Perinatal outcome^b^						
Live birth	753	62.91	262	75.07	1015	65.65
Alive within 7 days	618	51.63	187	53.58	805	52.07
Early neonate death	135	11.28	75	21.49	210	13.58
Stillbirth	444	37.09	87	24.93	531	34.35

^**a**^5 cases with missing birth weight were excluded; ^**b**^2 cases with missing perinatal outcome were excluded

## Discussion

By analyzing almost 10 million birth data in CBDMN, we identified an overall prevalence of 6.14/10,000 for CH during 2005 to 2012, which was significantly lower than the rate of 7.03/10,000 during 1996–2004 (*P* < 0.001) [14]. The overall rate in our study also appeared lower than those from previous studies conducted in mainland China (6.9–9.2/10,000) [11–13]. In comparison with some ICBDSR member programs with the same CH inclusion criterion, our prevalence was comparable to that in United Kingdom-Wales (6.43/10,000), higher than that in Canada (4.84/10,000), and lower than that in USA-Texas (7.88/10,000), Japan (7.81/10,000) and India (7.24/10,000) [21]. Notably, all the above-mentioned ICBDSR programs include cases or terminations of pregnancy less than 28 weeks of gestation, so the estimated prevalence could be higher than the current observation if CH cases of any gestational age were included in CBDMN. Previous study in California showed that Asian populations had the lowest CH prevalence, followed by Caucasians and Hispanics, and then Blacks [8]. The prevalence in the current study was higher than those reported in Caucasians in United States (5.4/10,000) [8] and several European regions (4.65/10,000) [7], but lower than those found in Sweden (6.6/10,000) [10] and Denmark (11.3/10,000) [9]. The considerable variations in previous studies may be explained by several factors such as ethnicity, socioeconomic factors, different case inclusion criterions, etc.

Similar to previous findings of studies in the United States [8], Sweden [10], as well as a few provinces of China [12, 13], a downward trend in CH prevalence was obtained in our study, which seemed to reverse an earlier upward trend from 1996 to 2004 from the same data source (CBDMN) [14]. In the United States a great decline was found in the prevalence of CH cases accompanied by NTDs, which was thought to be related with folic acid supplementation [23]. Since CH cases with NTDs were excluded in the current analysis, the inverse trend during recent 8-year period cannot be explained by specific interventions related to NTDs. Meanwhile, several studies in mainland China suggested that downward trend in CH prevalence was associated with the widely implementation of prenatal diagnosis service, which has been boosted by the *National Regulation on the Administration of Prenatal Diagnosis Techniques* since 2003. According to the regulation, pregnant women are recommended to have systemic ultrasound examination to screen for birth defects in the second trimester, and pregnancy affected by severe birth defects is legally allowed to be terminated through a standardized process. Reasonably, a portion of fetuses with severe hydrocephalus could be diagnosed and terminated before 28 weeks of gestation along with the widespread use of prenatal sonography, which might result in the decrease of CH prevalence to some extent. Nevertheless, the contribution of improved maternal nutrition status and prenatal health care should not be neglected.

In the current study, higher prevalence was found in both younger (<20 years) and advanced (≥ 35 years) maternal age groups. Data from Czech Republic showed that significantly higher risk of hydrocephalus was in women above 37 years of age [24]. A Denmark study indicated that mothers under the age of 20 were more likely to have offspring with isolated hydrocephalus than those aged 25 to 34 years [9]. The U-shaped maternal-age distribution of CH prevalence highlighted maternal age as a risk factor of CH though the underlying cause was still unknown.

There are substantial urban-rural differences in the residents’ education level, health literacy, occupational exposure, lifestyle, and access to health care in China [25]. The observed urban-rural variations in this study indicated that poor socioeconomic levels and individual health status were associated with increased CH risk. The male predominance in CH prevalence was consistent with previous studies [8–10], the underlying causes remain unclear although X-linked genetic factors can explain a small portion of cases [3]. Previous findings in the United States demonstrated that the CH prevalence of multiple births increased to three times as compared to singletons (23.08/10,000 vs 7.07/10,000) [26]. Similar result was obtained in our study (15.29/10,000 vs 5.99/10,000).

In our study, about one third of non-aborted hydrocephalus cases were low birth weight and preterm births. Given that CH cases secondary to intraventricular haemorrhage were excluded from the current analysis, the high portion of low birth weight and preterm births are likely due to impaired physical development. In another word, though preterm birth is risk factor of neonatal hydrocephalus, congenital hydrocephalus may increase the risk of preterms. In low resource setting, 47.8% of severe hydrocephalus and 55.6% of Dandy-walker syndrome cases died prior to 1 year of age [27]. However, the neonatal mortality rate in our study was lower (20.69%) due to the short monitoring period.

### Strengths and limitations

Based on a large-sample-size database with extensive geographic coverage and good data quality management, our findings represent the current status of CH in Chinese population, which can provide valuable references for future intervention in China as well as in other developing countries. Considering the possible ascertainment bias due to variation in the diagnosis between different member hospitals and the short monitoring period, the observed CH prevalence and mortality rate in this study might be underestimated. Due to data limitation, we can not make further cause-specific analysis.

## Conclusions

In conclusion, our analyses found a downward trend in birth prevalence of CH in China since 2005, and the prevalence varied significantly by maternal age, gender, residential area and plurality of pregnancy. The findings of relatively high prevalence and poor perinatal outcomes of infants with hydrocephalus are of great value for future study.

## Abbreviations

CBDMN: Chinese Birth Defects Monitoring Network
CH: Congenital hydrocephalus
CI: Confidence intervals
CSF: Cerebrospinal fluid
ENMR: Early neonatal mortality rate
ICBDSR: International Clearinghouse for Birth Defects Surveillance and Research
NTDs: Neural tube defects
PR: Prevalence ratios
